# Supplementary material for: Engineered tRNAs suppress nonsense mutations in cells and in vivo
Source: Nature. 2023 May 31;618(7966):842–8. doi: 10.1038/s41586-023-06133-1 (PMC10284701; doi:10.1038/s41586-023-06133-1)
Supplement: Supplementary file 1 — This file contains Supplementary Figs. 1a–c and 2 and Supplementary Tables 1–4. [file 41586_2023_6133_MOESM1_ESM.pdf]

nature portfolio

https://doi.org/10.1038/s41586-023-06133-1

Supplementary information

Engineered tRNAs suppress nonsense mutations in cells and in vivo

In the format provided by the authors and unedited

Engineered tRNAs suppress nonsense mutations in cells and in vivo

Suki Albers[1], Elizabeth C. Allen[2], Nikhil Bharti[1], Marcos Davyt[1], Disha Joshi[3,4], Carlos G. Perez-Garcia[2], Leonardo Santos[1], Rajesh Mukthavaram[2], Miguel Angel Delgado-Toscano[1], Brandon Molina[2], Kristen Kuakini[2], Maher Alayyoubi[2], Kyoung-Joo Jenny Park[2], Grishma Acharya[2], Jose A. Gonzalez[2], Amit Sagi[2], Susan E. Birket[5], Guillermo J. Tearney[6], Steven M. Rowe[5], Candela Manfredi[3,4], Jeong S. Hong[3,4], Kiyoshi Tachikawa[2], Priya Karmali[2], Daiki Matsuda[2], Eric J. Sorscher[3,4*], Pad Chivukula[2]*, Zoya Ignatova[1]*

[1]Institute of Biochemistry and Molecular Biology, University of Hamburg, 20146 Hamburg. Germany.

[2]Arcturus Therapeutics Inc., San Diego, CA 92121, USA.

[3]Department of Pediatrics, School of Medicine, Emory University, Atlanta, GA 30322, USA.

[4]Children's Healthcare of Atlanta, Atlanta, GA 30322, USA

[5]Pulmonary, Allergy, and Critical Care Medicine, University of Alabama at Birmingham, AL 35294, USA

[5]Wellman Center for Photomedicine, Massachusetts General Hospital, MA 02114, USA.

*Correspondence to: Zoya Ignatova, email: zoya.ignatova@uni-hamburg.de and Pad Chivukula, email: pad@arcturusrx.com and Eric Sorscher, email: esorscher@emory.edu

Table of contents

Supplementary Figure 1 (a-c) ……………………………………………………………3

Supplementary Figure 2 ……………………………………………………………7

Supplementary Table 1 ………………………………………………………….…8

Supplementary Table 2 …………………………………………………..………9

Supplementary Table 3 ……………………………..…………………………..10

Supplementary Table 4 ……………………………..…………………………..11

Supplementary Figures

Supplementary Figure 1. **Raw data of gel images from the capillary electrophoresis system (Jess, ProteinSimple).** Designated areas (red boxes) correspond to CFTR band C and are used in the main figures for quantification of CFTR protein expression.

a-c, Gels used in Fig. 3a to determine the efficacy of tS and tR variants in restoring expression of full-length CFTR(S466X), CFTR(R553X) or CFTR(R1162X) in CFBE41o^{-} cells

d, Gels used in Fig. 4b to determine the efficacy of tR or tRT5 efficacy alone or by co-treatment with NMD inhibitor (5 µM NMD14), or treatment with 10 µM and 30 µM PTC124 in 16HBEge$^{R1162X/-}$ (X represents UGA) and 16HBE14o^{-} expressing wild-type *CFTR* cells.

e, Gels used in Fig. 4c to determine the efficacy of tRT5 alone or by co-treatment with NMD inhibitor (5 µM NMD14) in hNE$^{R1162X/R1162X}$ and hNE expressing WT-CFTR.

a

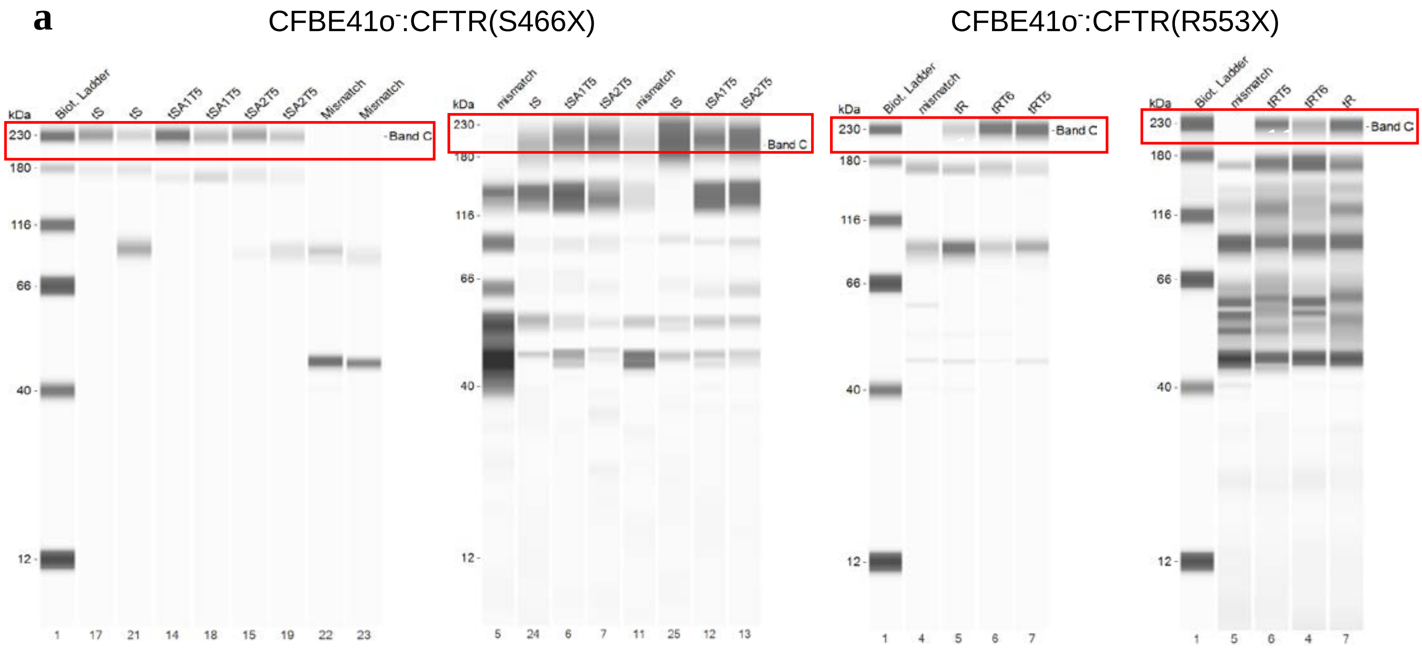

b

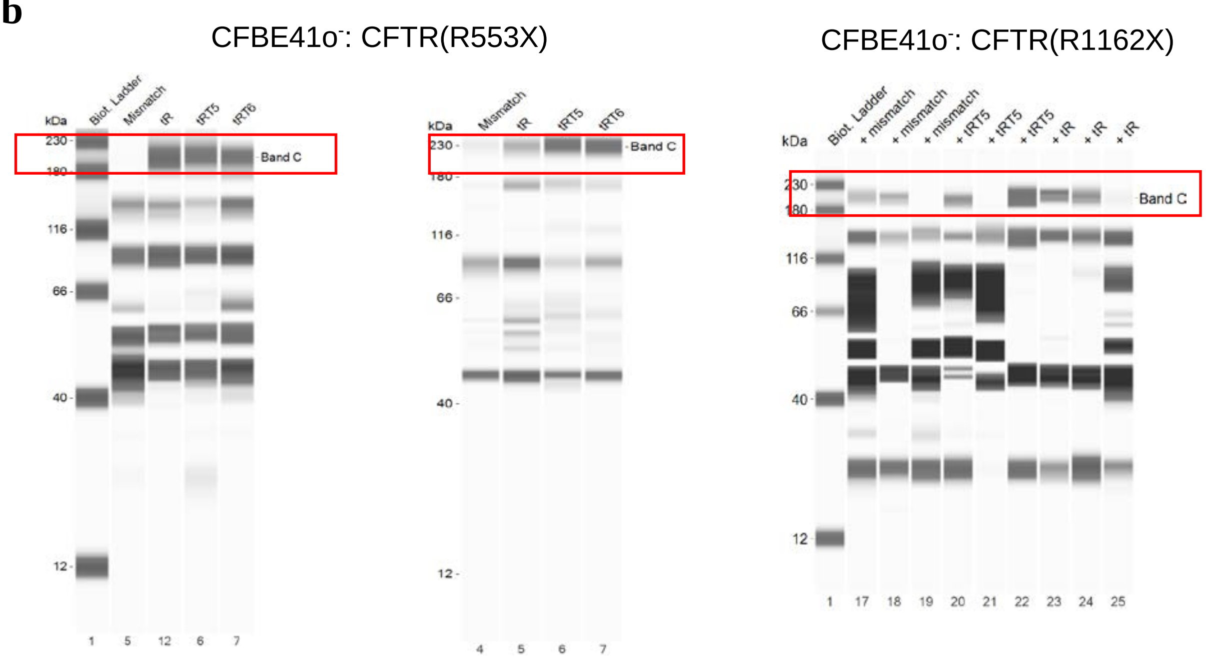
CFBE41o-: CFTR(R553X)
CFBE41o-: CFTR(R1162X)
kDa
Biot. Ladder
Mismatch
tR
tRT5
tRT6
Band C
230
180
116
66
40
12
+ mismatch
+ tRT5
+ tR

c

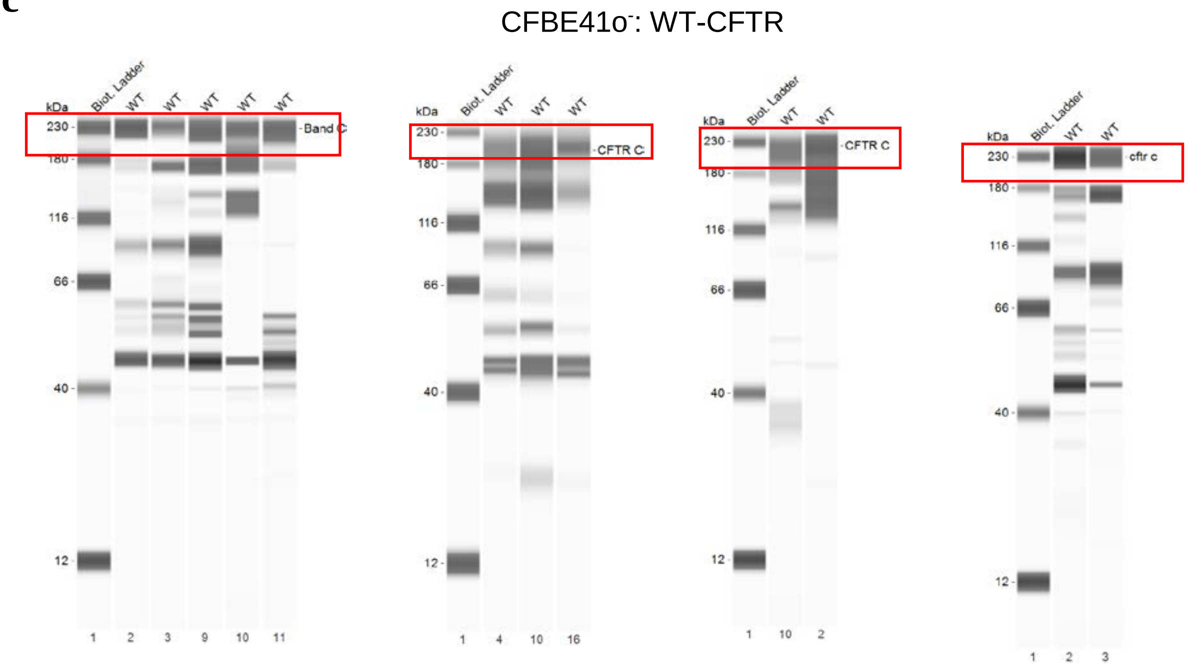
CFBE41o-: WT-CFTR
kDa
Biot. Ladder
WT
Band C
CFTR C
cftr c
230
180
116
66
40
12

d

16HBEge: CFTR(R1162X)

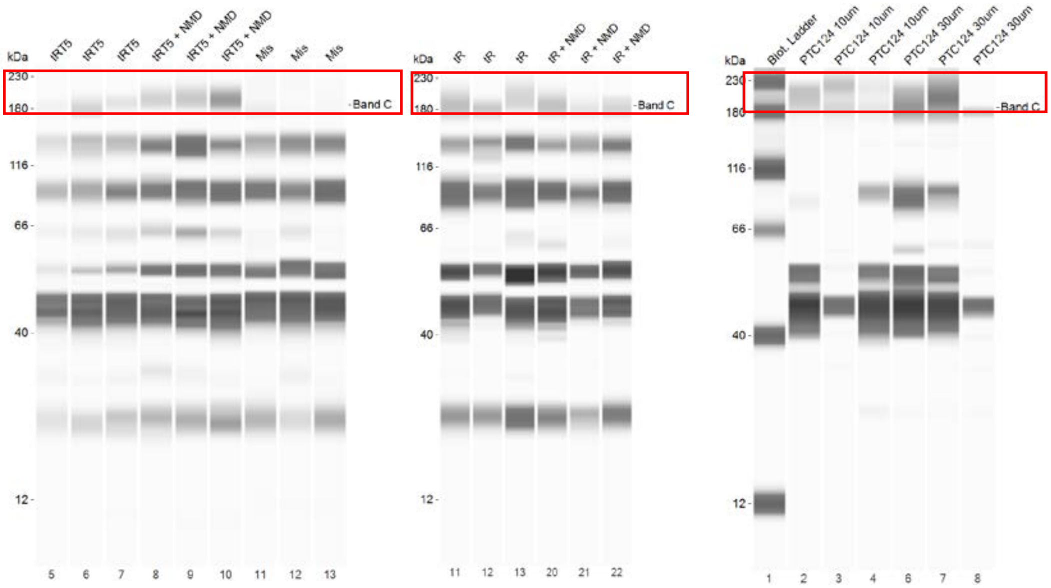

16HBE14o$^-$: WT-CFTR

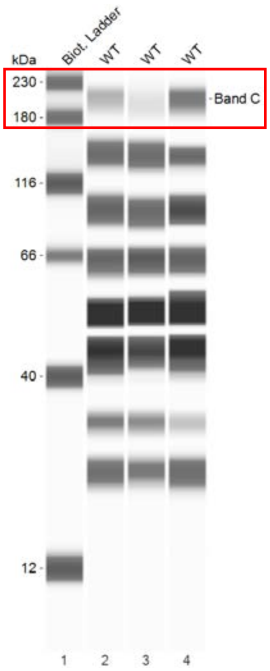

e

hNE: CFTR(R1162X)

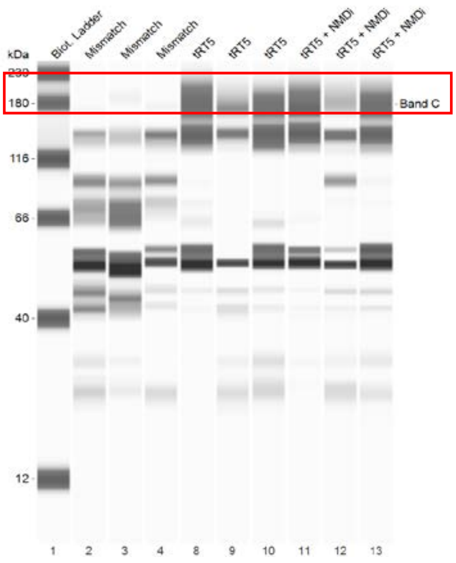

hNE: WT-CFTR

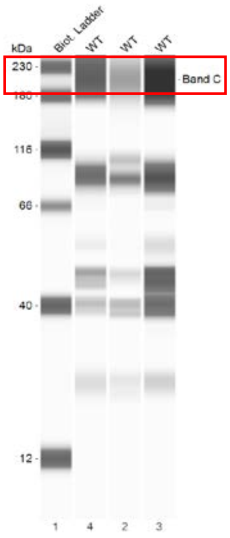

Supplementary Figure 2. Raw gel image. Cropped area (red box) is shown in Extended Data Fig. 2a.

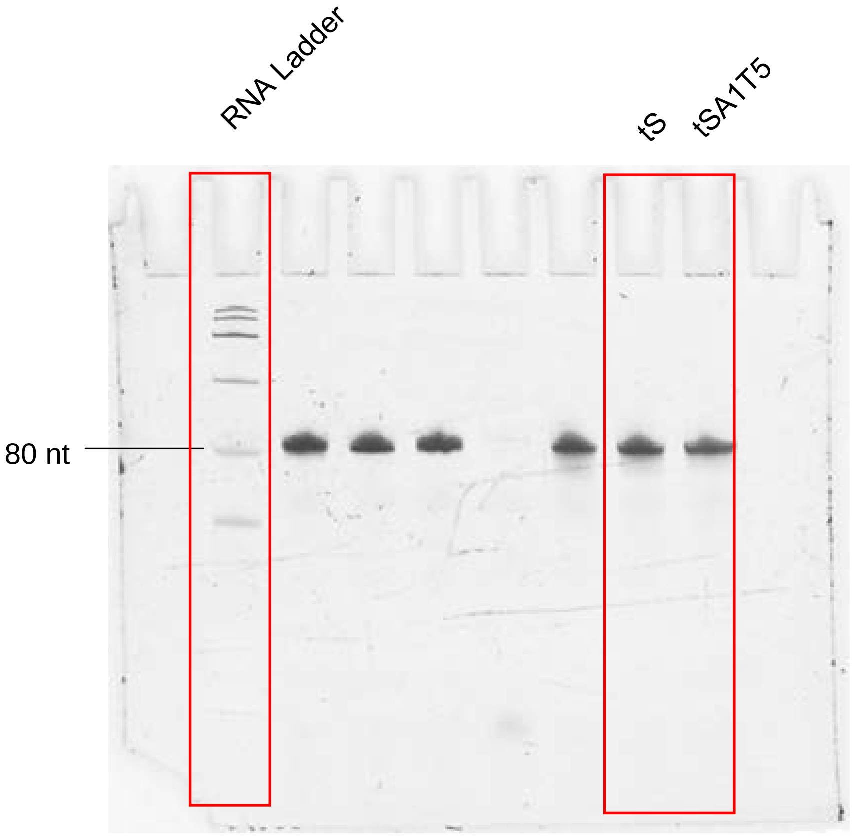

Supplementary Tables

Supplementary Table 1: Sequences of the tRNA variants. Substitutions in the anticodon are underlined, in the AC-stem and TΨC-stem highlighted in blue (according to Fig. 1a and Extended Data Fig. 1).

tRNA variant	tRNAscan-SE ID[a]	Sequence (5′-3′)
tRNA^{Ser} (AGA)	chr6.tRNA151	GUAGUCGUGGCCGAGUGGUUAAGGCGAUGGACUAGAAAUCCAUUGGGGUUUCCCCGCGCAGGUUCGAAUCCUGCCGACUACGCCA
tRNA^{Ser} (UGA)	chr6.tRNA53	GUAGUCGUGGCCGAGUGGUUAAGGCGAUGGACUUGAAAUCCAUUGGGGUUUCCCCGCGCAGGUUCGAAUCCUGCCGACUACGCCA
tS		GUAGUCGUGGCCGAGUGGUUAAGGCGAUGGACUUCAAAUCCAUUGGGGUUUCCCCGCGCAGGUUCGAAUCCUGCCGACUACGCCA
tSA1		GUAGUCGUGGCCGAGUGGUUAAGGCGAUGGUCUUCAAAACCAUUGGGGUUUCCCCGCGCAGGUUCGAAUCCUGCCGACUACGCCA
tSA2		GUAGUCGUGGCCGAGUGGUUAAGGCGGCAGGCUUCAAACCUGUUGGGGUUUCCCCGCGCAGGUUCGAAUCCUGCCGACUACGCCA
tST1		GUAGUCGUGGCCGAGUGGUUAAGGCGAUGGACUUCAAAUCCAUUGGGGUUUCCCCGCGCCGGUUCGAAUCCGGCCGACUACGCCA
tST2		GUAGUCGUGGCCGAGUGGUUAAGGCGAUGGACUUCAAAUCCAUUGGGGUUUCCCCGCCCGGGUUCGAAUCCCGGCGACUACGCCA
tST3		GUAGUCGUGGCCGAGUGGUUAAGGCGAUGGACUUCAAAUCCAUUGGGGUUUCCCCGCAGGCCUUCGAAUGGCCUCGACUACGCCA
tST4		GUAGUCGUGGCCGAGUGGUUAAGGCGAUGGACUUCAAAUCCAUUGGGGUUUCCCCGCGCGGGUUCGAAUCCCGCCGACUACGCCA
tST5		GUAGUCGUGGCCGAGUGGUUAAGGCGAUGGACUUCAAAUCCAUUGGGGUUUCCCCGCACGGGUUCGAAUCCCGUCGACUACGCCA
tST6		GUAGUCGUGGCCGAGUGGUUAAGGCGAUGGACUUCAAAUCCAUUGGGGUUUCCCCGCGCGGGUUCGAAUCCCGUCGACUACGCCA
tSA1T4		GUAGUCGUGGCCGAGUGGUUAAGGCGAUGGUCUUCAAAACCAUUGGGGUUUCCCCGCGCGGGUUCGAAUCCCGCCGACUACGCCA
tSA1T5		GUAGUCGUGGCCGAGUGGUUAAGGCGAUGGUCUUCAAAACCAUUGGGGUUUCCCCGCACGGGUUCGAAUCCCGUCGACUACGCCA
tSA1T6		GUAGUCGUGGCCGAGUGGUUAAGGCGAUGGUCUUCAAAACCAUUGGGGUUUCCCCGCGCGGGUUCGAAUCCCGUCGACUACGCCA
tSA2T5		GUAGUCGUGGCCGAGUGGUUAAGGCGGCAGGCUUCAAACCUGUUGGGGUUUCCCCGCACGGGUUCGAAUCCCGUCGACUACGCCA
tRNA^{Arg} (UCU)	chr9.tRNA5 chr11.tRNA2	GGCUCUGUGGCGCAAUGGAUAGCGCAUUGGACUUCUAAUUCAAAGGUUGUGGGUUCGAGUCCCACCAGAGUCGCCA
tR		GGCUCUGUGGCGCAAUGGAUAGCGCAUUGGACUUCAAAUUCAAAGGUUGUGGGUUCGAGUCCCACCAGAGUCGCCA
tRA2		GGCUCUGUGGCGCAAUGGAUAGCGCAGCAGGCUUCAAACCUGUAGGUUGUGGGUUCGAGUCCCACCAGAGUCGCCA
tRT5		GGCUCUGUGGCGCAAUGGAUAGCGCAUUGGACUUCAAAUUCAAAGGUUACGGGUUCGAGUCCCGUCAGAGUCGCCA
tRT6		GGCUCUGUGGCGCAAUGGAUAGCGCAUUGGACUUCAAAUUCAAAGGUUGCGGGUUCGAGUCCCGUCAGAGUCGCCA
tRT7		GGCUCUGUGGCGCAAUGGAUAGCGCAUUGGACUUCAAAUUCAAAGGUUAUGGGUUCGAGUCCCAUCAGAGUCGCCA
tRT8		GGCUCUGUGGCGCAAUGGAUAGCGCAUUGGACUUCAAAUUCAAAGGUUGCGGGUUCGAGUCCCGCCAGAGUCGCCA
tRT9		GGCUCUGUGGCGCAAUGGAUAGCGCAUUGGACUUCAAAUUCAAAGGUUGUGGGUUCGAGUCCCAUCAGAGUCGCCA
tRA2T5		GGCUCUGUGGCGCAAUGGAUAGCGCAGCAGGCUUCAAACCUGUAGGUUACGGGUUCGAGUCCCGUCAGAGUCGCCA
tRNA^{Gly} (UCC)	chr17.tRNA9	GCGUUGGUGGUAUAGUGGUAAGCAUAGCUGCCUUCCAAGCAGUUGACCCGGGUUCGAUUCCCGGCCAACGCACCA
tG1		GCGUUGGUGGUAUAGUGGUAAGCAUAGCUGCCUUCAAAGCAGUUGACCCGGGUUCGAUUCCCGGCCAACGCACCA
tG1T5		GCGUUGGUGGUAUAGUGGUAAGCAUAGCUGCCUUCAAAGCAGUUGACACGGGUUCGAUUCCCGUCCAACGCACCA
tG1T6		GCGUUGGUGGUAUAGUGGUAAGCAUAGCUGCCUUCAAAGCAGUUGACGCGGGUUCGAUUCCCGUCCAACGCACCA
tRNA^{Gly} (UCC)	chr19.tRNA2	GCGUUGGUGGUAUAGUGGUUAGCAUAGCUGCCUUCCAAGCAGUUGACCCGGGUUCGAUUCCCGGCCAACGCACCA
tG2		GCGUUGGUGGUAUAGUGGUUAGCAUAGCUGCCUUCAAAGCAGUUGACCCGGGUUCGAUUCCCGGCCAACGCACCA
tG2T5		GCGUUGGUGGUAUAGUGGUUAGCAUAGCUGCCUUCAAAGCAGUUGACACGGGUUCGAUUCCCGUCCAACGCACCA
tG2T6		GCGUUGGUGGUAUAGUGGUUAGCAUAGCUGCCUUCAAAGCAGUUGACGCGGGUUCGAUUCCCGUCCAACGCACCA
mis-match tRNAs		GUAGUCGUGGCCGAGUGGUUAAGGCGAUGGACUUUAAAUCCAUUGGGGUUUCCCCGCGCAGGUUCGAAUCCUGCCGACUACGCCA
		GUAGUCGUGGCCGAGUGGUUAAGGCGAUGGACUCUAAAUCCAUUGGGGUUUCCCCGCGCAGGUUCGAAUCCUGCCGACUACGCCA
		GUAGUCGUGGCCGAGUGGUUAAGGCGAUGGUCUUUAAAACCAUUGGGGUUUCCCCGCACGGGUUCGAAUCCCGUCGACUACGCCA
		GUAGUCGUGGCCGAGUGGUUAAGGCGAUGGUCUCUAAAACCAUUGGGGUUUCCCCGCACGGGUUCGAAUCCCGUCGACUACGCCA
		GUAGUCGUGGCCGAGUGGUUAAGGCGGCAGGCUCUAAACCUGUUGGGGUUUCCCCGCACGGGUUCGAAUCCCGUCGACUACGCCA
		GUAGUCGUGGCCGAGUGGUUAAGGCGAUGGACUUGUUAAUCCAUUGGGGUUUCCCCGCGCAGGUUCGAAUCCUGCCGACUACGCCA

[a]tRNAscan IDs are from the tRNA data base (GtRNAdb), http://gtrnadb.ucsc.edu/

Supplementary Table 2: Summary of the sequence context of pathologic PTCs used in this study. Nucleotide and amino sequence of human PTCs are incorporated downstream of the start codon of *FLuc* (PTC-FLuc variants). The PTC is underlined and is flanked by seven codons each site.

Disease	Protein	Mutation	PTC	Nucleotide and amino acid sequence of PTC contexts
CLN2	TPP1	R208X	UGA	ACCCCCTCTGTGATCCGTAAGTGATACAACTTG ACCTCACAAGAC
			UAG	ACCCCCTCTGTGATCCGTAAGTAGTACAACTTG ACCTCACAAGAC
			UAA	ACCCCCTCTGTGATCCGTAAGTAATACAACTTG ACCTCACAAGAC
				-T--P--S--V--I--R--K--X--Y--N--L--T--S--Q--D-
CF	CFTR	S466X	UGA	TCCACTGGAGCAGGCAAGACTTGACTTCTAATGATGATTATGGGA
			UAG	TCCACTGGAGCAGGCAAGACTTAGCTTCTAATGATGATTATGGGA
			UAA	TCCACTGGAGCAGGCAAGACTTAACTTCTAATGATGATTATGGGA
				-S--T--G--A--G--K--T--X--L--L--M--M--I--M--G-

Supplementary Table 3. LUNAR1 and LUNAR2 nanoparticulate encapsulations.

LUNAR formulations	Particle size [nm]	Polydispercity index, PDI	% Encapsulation
LUNAR 1	83.45	0.133	96.4
LUNAR 2	75.61	0.168	99.4

Supplementary Table 4. Total number of transcripts detected in the ribosome profiling data sets.

Murine liver			
	Treated with tS Replicate #1	Treated with tS Replicate #2	Control
UAA	2791	2636	2504
UAG	2552	2424	2295
UGA	5747	5441	5109
Total	10900	10501	9908

Murine lungs			
	Treated with tSA1T5 Replicate #1	Treated with tSA1T5 Replicate #2	Control
UAA	3325	2580	3080
UAG	3012	2338	2778
UGA	6690	5259	6215
Total	13027	10117	12073
