# Supplementary material for: Engineered tRNAs suppress nonsense mutations in cells and in vivo
Source: Nature. 2023 May 31;618(7966):842–8. doi: 10.1038/s41586-023-06133-1 (PMC10284701; doi:10.1038/s41586-023-06133-1)
Supplement: Supplementary file 2 — Reporting Summary [file 41586_2023_6133_MOESM2_ESM.pdf]

nature portfolio

Corresponding author(s): Zoya Ignatova

Last updated by author(s): Apr 4, 2023

Reporting Summary

Nature Portfolio wishes to improve the reproducibility of the work that we publish. This form provides structure for consistency and transparency in reporting. For further information on Nature Portfolio policies, see our Editorial Policies and the Editorial Policy Checklist.

Statistics

For all statistical analyses, confirm that the following items are present in the figure legend, table legend, main text, or Methods section.

n/a	Confirmed	
☐	☒	The exact sample size (n) for each experimental group/condition, given as a discrete number and unit of measurement
☐	☒	A statement on whether measurements were taken from distinct samples or whether the same sample was measured repeatedly
☐	☒	The statistical test(s) used AND whether they are one- or two-sided<br>*Only common tests should be described solely by name; describe more complex techniques in the Methods section.*
☒	☐	A description of all covariates tested
☐	☒	A description of any assumptions or corrections, such as tests of normality and adjustment for multiple comparisons
☐	☒	A full description of the statistical parameters including central tendency (e.g. means) or other basic estimates (e.g. regression coefficient) AND variation (e.g. standard deviation) or associated estimates of uncertainty (e.g. confidence intervals)
☐	☒	For null hypothesis testing, the test statistic (e.g. F, t, r) with confidence intervals, effect sizes, degrees of freedom and P value noted<br>*Give P values as exact values whenever suitable.*
☒	☐	For Bayesian analysis, information on the choice of priors and Markov chain Monte Carlo settings
☒	☐	For hierarchical and complex designs, identification of the appropriate level for tests and full reporting of outcomes
☒	☐	Estimates of effect sizes (e.g. Cohen's d, Pearson's r), indicating how they were calculated

Our web collection on statistics for biologists contains articles on many of the points above.

Software and code

Policy information about availability of computer code

Data collection	Bulk activity (luciferase/renilla) measurements: Spark microplate reader, SparkControl Version 2.3 (Tecan).<br>Mass spectrometry: 2D nano PRM LC-MS/MS by Jade Bio, Inc (San Diego, CA)<br>qRT-PCR: T professional thermocycler (Biometra)<br>Immunoblots: Jess capillary electrophoresis system (Jess, ProteinSimple)<br>Cell fluorescence imaging: EVOS Cell Imaging Systems (Thermo Fisher Scientific)<br>Live animal bioluminescence imaging: IVIS Lumina III (Perkin Elmer, USA)<br>Ussing chamber for CFTR channel activity measurements (Psysiologic Instruments, Inc.)
Data analysis	Immunoblot quantification: Compass for SW Version 6.0.0 (Jess, ProteinSimple)<br>Live animal bioluminescence imaging quantification: Living Image Software, version 4.7.4. (Perkin Elmer)<br>CFTR channel activity - ACQUIRE & ANALYZE v.2.3 package provided by Ussing chamber vendor (Physiologic Instruments, Inc.) and measures current, voltage, conductance and rsistance from up to 8 tissues simultaneously.<br>µOCT measuremnts - Image J (version 1.49)

For manuscripts utilizing custom algorithms or software that are central to the research but not yet described in published literature, software must be made available to editors and reviewers. We strongly encourage code deposition in a community repository (e.g. GitHub). See the Nature Portfolio guidelines for submitting code & software for further information.

Data

Policy information about availability of data

All manuscripts must include a data availability statement. This statement should provide the following information, where applicable:

- Accession codes, unique identifiers, or web links for publicly available datasets
- A description of any restrictions on data availability
- For clinical datasets or third party data, please ensure that the statement adheres to our policy

All data are available in the main text or the supplementary materials.
Ribosome profiling data (Ribo-seq) from mouse organs and human CFBE41o- cells and tRNA microarray generated in this study are available at the Gene Omnibus (GEO) under the accession numbers GSE191048, GSE 192623 and GSE205660, respectively. For mapping of the Ribo-seq data sets human (GRCh38) and mouse (GRCm38) reference genomeswere used, respectively.

Field-specific reporting

Please select the one below that is the best fit for your research. If you are not sure, read the appropriate sections before making your selection.

☒ Life sciences ☐ Behavioural & social sciences ☐ Ecological, evolutionary & environmental sciences

For a reference copy of the document with all sections, see nature.com/documents/nr-reporting-summary-flat.pdf

Life sciences study design

All studies must disclose on these points even when the disclosure is negative.

Sample size	Sample size is stated in figure legends. For readthrough efficiency, 3 mice were used and for Ribo-seq 2 mice. Experiments were designed to detect differences greater than 20% at a significance of p<0.05.
Data exclusions	No data were excluded.
Replication	Experiments were reproduced in multiple (2-10) independent biological replicates each time using independent experimental methods. In each figure legends the number of independent biological replicates is stated. The majority of the assays wered cunducted three times, which demonstrates the high reproducibility of the data.
Randomization	Animals were chosen randomly and assigned either as control group or for treatment. For immunofluorescence and immunohistochemistry, the fields of view were randomly chosen. For other experiments, where the samples were not randomly assigned, there was no need for randomization, because all samples were independently measured in a controlled manner
Blinding	For the majority of the experiments no blinding was performed as blinding is not possible or not applicable for the experiments; only in the µOCT assays the geometric measurement of the corresponding layers was perfomed by an investigator blinded to the treatment. The in vivo reserachers were not blinded, as they performed the treatments. In cell culture experiments, blinding is not applicable because the researchers need to verify samples and controls for each experiment.<br>However, in almost all experiemnts, a second researcher confirmed the results.

Reporting for specific materials, systems and methods

We require information from authors about some types of materials, experimental systems and methods used in many studies. Here, indicate whether each material, system or method listed is relevant to your study. If you are not sure if a list item applies to your research, read the appropriate section before selecting a response.

Materials & experimental systems

n/a	Involved in the study	
☐	☒	Antibodies
☐	☒	Eukaryotic cell lines
☒	☐	Palaeontology and archaeology
☐	☒	Animals and other organisms
☒	☐	Human research participants
☒	☐	Clinical data
☒	☐	Dual use research of concern

Methods

n/a	Involved in the study	
☒	☐	ChIP-seq
☒	☐	Flow cytometry
☒	☐	MRI-based neuroimaging

Antibodies

Antibodies used	1) primary rabbit polyclonal RFP antibody (#600-401-379, Lot #42896, dilution 1:800, Rockland Antibody)

Antibodies used	2) mouse monoclonal FoxJ1 (#14-9965-82, Lot #2262272, dilution 1:100, ThermoFisher Scientific)<br>3) rabbit polyclonal Muc5B (#PA5-82342, Lot # VL31536898, dilution 1:600, ThermoFisher Scientific)<br>4) anti CFTR-NBD2 antibody (#596, Lot #596TJ03182012, 1:100 dilution, John R. Riordan and Tim Jensen, University of North Carolina, Chapel Hill, USA)<br>5) Donkey anti mouse AF488 (#A-21202, dilution 1:1000 ThermoFisher Scientific).<br>6) Donkey anti rabbit AF555 (#A-21428, dilution 1:1000, ThermoFisher Scientific)
Validation	For Jess-Immunoblot experiments, we tested different dilutions in the range 1:10-1:250 which is recommended by the manufacturer. The optimal dilution was 1:100.<br><br>The primary rabbit polyclonal RFP antibody (#600-401-379, Rockland Antibody) was internally validated for immunohistochemistry on transgenic models expressing TdTomato protein and also by the vendor. Used in Fig. 2 and Extended Data Fig. 7. Antibody profile webpage: https://rockland-inc.com/store/Antibodies-to-GFP-and-Antibodies-to-RFP-600-401-379-O4L_24299.aspx<br><br>For mouse monoclonal FoxJ1 (#14-9965-82, ThermoFisher Scientific) and rabbit polyclonal Muc5B (#PA5-82342, ThermoFisher Scientific) the vendors provided validation reports for tests in different organisms. The suitability of the rabbit polyclonal Muc5B for immunohistochemistry on mouse tissues was tested internally. Both antibodies were used in Fig. 2. Vendors antibody profile webpages: Muc5B - i. https://www.thermofisher.com/antibody/product/MUC5B-Antibody-Polyclonal/PA5-82342, and FoxJ1 - i. https://www.thermofisher.com/antibody/product/FOXJ1-Antibody-clone-2A5-Monoclonal/14-9965-82<br><br>Donkey anti-mouse AF488: https://www.thermofisher.com/antibody/product/Donkey-anti-Mouse-IgG-H-L-Secondary-Antibody-Polyclonal/R37114<br><br>Donkey anti rabbit AF555: https://www.thermofisher.com/antibody/product/Donkey-anti-Rabbit-IgG-H-L-Highly-Cross-Adsorbed-Secondary-Antibody-Polyclonal/A-31572

Eukaryotic cell lines

Policy information about cell lines

Cell line source(s)	Human Hep3B (HB-8064) and murine Hepa 1-6 (CRL-1830) were obained form ATCC/DSM.<br>HEK293XL TLR7 and TLR8 cells from Invivogen.<br>Cre-lox HEK293T (CRL-3216) reporter cell line was received from Dr. Ramon Trelles (Assoc. Director of Drug Discovery at Arcturus Therpaeutics).<br>CFBE41o- laboratory collection; originally received from Dr. Karl Kunzelmann, University of Regensburg, Germany) and Dr. Dieter Gruenert (University of California, San Francisco, CA).<br>Stably transfected R553X-CFTR and R1162X-CFTR FRT cell lines were created by Jeong S. Hong, a co-author of this study.<br>16HBEge cells (R1162X/-): received from the Cystic Fibrosis Foundation Therapeutics Lab.<br>16HBE14o- WT-CFTR obtained from Dr. Michael Lalk (Univ. Greifswald, Germany)<br>Primary human nasal epithelial cells (hNE R1162X/R1162X): received from the Cystic Fibrosis Foundation Therapeutics Lab.<br>Primary human nasal epithelial cells fron non-CF individual expressing wildtype CFTR: received from the Cystic Fibrosis Foundation Therapeutics Lab.
Authentication	For the purchased cell lines the vendor provided the authentication protocol and no additional authentification was used.<br>CFBE41o- was tested for the deletion of WT-CFTR (by sequencing and immunoblot).<br>The presence of R553X mutation in CFTR and the CFTR integration were verified by sequencing.
Mycoplasma contamination	Cell lines directly purchased from ATCC/DSM for the study arrived with a certificate for mycoplasma test and were not tested at arrival. All other cells were tested at arrival for mycoplasma contamination using Venor GeM PCR-based detection kit (Merck) and the tests were negative. Tested aliquots at low passage number were stored in liquid nitrogen. Cell lines in culture are regularly (every 6 mo. or by indication) tested for mycoplasma contamination and they all tested negative.
Commonly misidentified lines<br>(See ICLAC register)	No commonly misidentified cell lines were used.

Animals and other organisms

Policy information about studies involving animals; ARRIVE guidelines recommended for reporting animal research

Laboratory animals	10-weeks old female Balb/C mice were purchased from Charles River Laboratories and 7-8 weeks old female B6.Cg-Gt(ROSA)26Sortm14(CAG-tdTomato)Hze/J were purchased from Jackson Laboratories. All mice were purpose bred and experimentally naïve at the start of the study. Mice were housed 5 per cage in a pathogen-free environment in Innovive disposable IVC rodent caging system with a 12h light/dark cycle, at temperature between 19-22°C and humidity 50-60%. Ad libitum access to standard diet (2018, Global 18% protein rodent diet from Envigo+++, San Diego, CA, USA) and pre-filled acidified water from Innovive (pH 2.5-3.0) were used throughout the study period. The bedding material was hardwood chips (Sani-Chips, Cat# 7115, Envigo++++, CA, USA) and cages were changed biweekly.
Wild animals	No wild animals were used in this study.
Field-collected samples	No field collected samples were used in this study.
Ethics oversight	All studies were performed at Arcturus Therapeutics in accordance with the animal use protocols and policies approved by the Institutional Animal Care and Use Committee (IACUC) by an AAALAC approved vendor Explora BioLabs. The IACUC issuing organization is Explora BioLabs, protocol number EB17-004-003 from 2/1/17 and latest amendment from 6/17/21.

Note that full information on the approval of the study protocol must also be provided in the manuscript.
